# The Concept of "Heart Failure with Preserved Ejection Fraction": Time for a Critical Reappraisal

**DOI:** 10.31083/j.rcm2407202

**Published:** 2023-07-14

**Authors:** Gabriele Fragasso

**Affiliations:** ^1^Heart Failure Clinic, Istituto Scientifico San Raffaele, 20132 Milano, Italy

**Keywords:** heart failure, preserved ejection fraction, HFpEF, HFrEF, pathophysiology, myocardial fibrosis, atrial fibrillation, arterial hypertension, coronary artery disease, ischemia, drug therapy, phenotype, elderly

## Abstract

Heart failure with preserved ejection fraction (HFpEF) is frequently observed in elderly physically deconditioned subjects, mainly women with hypertension, obesity, glucose intolerance/diabetes, atrial fibrillation, anaemia, coronary artery disease, chronic pulmonary disease, and chronic renal insufficiency. In practice, these conditions represent the majority of cardiac diseases we deal with in our daily clinical practice. For this reason, the HFpEF disease does not exist as a single entity and, as such, no specific unifying therapy could be found. New classification attempts still do not consider the multifaceted aspect of the HF syndrome and appear rather as an artefactual attempt to categorize a condition which is indeed not categorizable. The aim of the present article is to critically review the construction of the concept of the HFpEF syndrome and propose the return of a pathophysiological approach in the evaluation and treatment of patients. Considering the huge economic efforts employed up to date to run awfully expensive trials and research in this field, it is time to call action and redirect such resources towards more specific pathophysiological classifications and potential specific therapeutic targets.

## 1. Introduction

The clinical enigma of heart failure (HF) without apparent evidence of HF has been fascinating the medical/cardiological community for the last 40 years [1, 2, 3]. Initially, HF is classified as “diastolic” (preserved ejection fraction) or “systolic” (reduced ejection fraction), but this nomenclature became controversial [4, 5] and the term “HF with preserved ejection fraction” (HFpEF) rather than “diastolic HF” was used [6]. The guidelines for the diagnosis and treatment of acute and chronic HF issued by the European Society of Cardiology (ESC) in 2016 have added a new class of HF, namely HF with mildly reduced (mid-range) ejection fraction (HfmrEF) while HFpEF indicated a clinical form of HF in which the ejection fraction is normal [7]. HFpEF accounts for one-half of all patients with HF and its natural history has been reported to be as ominous as the prognosis of patients suffering of systolic HF [8, 9, 10]. Nevertheless, while patients with chronic HF and reduced ejection fraction (HFrEF) have been shown to respond to a “one size fits all” approach, with several drugs and devices shown to improve outcome, clinical drug trials in HFpEF patients have been disappointing, and no treatment has improved survival in this group of patients. The reasons for these “fiascos” would have been interpreted as dependent on the heterogeneity of patient population recruited in the drug trials. In fact, many predisposing causes, often coexisting, can contribute to the development of HFpEF and therefore, in such context, the “one size fits all” approach can be reductive and inconclusive.

The aim of the present article is to critically review the concept of the HFpEF syndrome and propose the return of a pathophysiological approach in the evaluation and treatment of patients. Published literature is extensive and therefore the reported articles are limited to just a few principal ones.

## 2. Definition of Heart Failure

HF is defined as inability of the heart to keep up with the demands on it and, specifically, failure of the heart to pump blood with normal efficiency. When this occurs, the heart is unable to provide adequate blood flow to other organs such as the brain, liver and kidneys. A definite cardiac dysfunction is implicit in the term “failure”, therefore we must face compensated and decompensated HF. A number of patients with severe systolic HF, never decompensate. Conversely the so called HFpEF, where either only minor structural abnormalities or none are present and, therefore, HF is not the case, eventually becomes HF when decompensated.

The only systematic attempt to define HFpEF is provided by the ESC [7]. Elevation of natriuretic peptides are not considered a stringent criterion, since these markers are likely to be difficult to interpretate in the early stages of HFpEF. Natriuretic peptides are also influenced by age, female gender, presence of obesity, atrial fibrillation, anaemia, hypertrophy, pulmonary disease, and renal insufficiency [11, 12, 13].

More recently, to comply with the search for a therapeutic strategy in HFpEF, the introduction of the concept of HFmrEF has become necessary [7]. In fact, as already stated, the treatment of patients with heart insufficiency/incompetence without evidence of failure has been consistently unworthy and, therefore, the concept of HFmrEF is a partial convergence on the concept that in the context of heart failure you must have some real proof of heart failure, not just predisposing factors. On the other hand, you may have a heart unable to undergo certain challenges, then failing to maintain a correct output and determining decompensation. Following the resolution of the challenge and return of cardiac output to normality, can we label that patient as having HF, even with the presence of preserved biventricular function? The patient would certainly be classified as a high-risk patient, as he/she should have been diagnosed before. We already treat these patients aggressively for the control of their risk factors and conditions predisposing to potential decompensation.

Finally, when we consider HFpEF, it is generally only the left ventricle (LV) that is considered. However, the right ventricle (RV) plays as much an important role as the left one and therefore this role should not be minimized. Despite these elemental concepts are the base of common pathophysiology knowledge, they are regularly ignored. These concepts will be discussed in detail in the following paragraphs.

**Epidemiology of HFpEF.** Since the initial description, the proportion of patients with the diagnosis of HFpEF has steadily increased. In 2015, Gerber and colleagues [8] confirmed this trend, reporting that the overall incidence of HF declined of 37.5% between the years 2000 and 2010. Although the incidence of HF declined for both HFpEF and HFrEF, the declines were greater for HFrEF (–45%) than for HFpEF (–28%). HF ascertainment usually relies on diagnostic codes often without additional medical record abstraction and adjudication. Among confounding factors, it is interesting to note that diagnostic codes are influenced also by nonmedical factors, such as reimbursement incentives [10]. Nevertheless, the diagnosis of acute heart failure/decompensation should certainly be (but is) an easy one. Guidelines are quite clear and medical students/junior doctors learn very quickly how to diagnose and treat it in the acute phase. Then, on discharge, most of these patients will be labelled as heart failure patients, and heart failure, regardless of biventricular function, will become the culprit diagnosis these patients will indefinitely live with. An acute episode of cardiac decompensation may arise from many pathologic conditions, which operate on different individual specific phenotypes. This is what we observe in the vast majority of our steadily increasing elderly population, the older the greater the chance to decompensate in one or, usually as a summation circuit, more organ districts. Should we label all these people as heart failure patients? Or are they just elderly who develop frailty and decreased organ reserve?

There are also examples of acute cardiac decompensation without “heart failure”. As an extreme example, let us consider the athlete practicing strenuous exercise in difficult climate conditions, where the superimposition of dehydration on hyperthermia during exercise in the heat causes an inability to maintain cardiac output and blood pressure that makes the dehydrated athlete unable to cope with hyperthermia [14]. Additionally, these kinds of athletes present various degrees of LV hypertrophy (LVH) in otherwise super healthy hearts. In these contexts, LVH is considered as a physiological adaptation to strenuous exercise. Yet LVH is there, and whenever other patho/physiological conditions intervene, the so-called “physiological” LVH may contribute to decompensation. This is acute cardiac decompensation, yet that patient should not be considered as having chronic HFpEF. Nevertheless, according to the present diagnostic definitions (an episode of acute cardiac decompensation in a patient with an increased LV wall thickness), we should consider this athlete as a patient with chronic HfpEF, would this be appropriate?

**Echocardiographic features in patients with HFpEF.** Diastolic dysfunction, left atrial enlargement, and pulmonary hypertension are frequent in patients with HFpEF, as evidenced by the echocardiography sub study of the PARAGON-HF (Prospective Comparison of angiotensin receptor neprilysin inhibitors (ARNI) With ARB Global Outcomes in HF With Preserved Ejection Fraction) study [15]. LVH, elevated left- and right-sided pressures, and RV enlargement were predictive of incident heart failure hospitalization or cardiovascular death, while Doppler-based diastolic measures (E wave, TDI $e^{\prime }$, and E/$e^{\prime }$ ratio) were most strongly associated with risk for HF hospitalization, and systemic venous congestion was particularly relevant for risk of recurrent HF hospitalizations.

Pre-existing diastolic dysfunction in the presence of precipitating factors may result in acute systolic dysfunction and pulmonary oedema. Prevalence of isolated LV diastolic dysfunction has been reported to be around one-third of all HF cases, with an increasing prevalence in the elderly population [16]. Patients with HF and isolated diastolic dysfunction present clinical symptoms, quality of life, readmission and 6-month mortality rates like patients with prevalent LV systolic dysfunction [17]. In a prospective HFpEF registry, only patients with moderate to severe diastolic dysfunction showed poor prognosis over short term follow-up [18]. A more recent retrospective analysis among elderly individuals with heart failure has shown that specific parameters such as wall thickness, atrial dimensions, NT-proBNP, and pulmonary vein velocities better predicted HF readmission in HFpEF than HFrEF; further echocardiographic structural and diastolic variables augmented prediction of HF readmission compared with comorbidities alone, regardless of LVEF, though predictive accuracy remained modest [19].

**LV systolic function in HFpEF at the time of decompensation.** In patients with decompensated HFpEF (i.e., acute decompensation), clinical signs and symptoms and diagnostic tests are likely to be abnormal. In stable patients with exertional symptoms only, the diagnosis of HFpEF becomes doubtful. In these patients with echocardiographic normal EF, the clinical examination, chest X-ray, electrocardiogram and natriuretic peptides may be normal.

In fact, in a recent analysis in hospitalized participants of the ESC-Heart Failure Association (HFA) EURObservational Research Programme (EORP) HF Long-Term Registry, who evidenced ejection fraction (EF) ≥50% during index hospitalization, acute HFpEF diagnosis could be assessed in only a quarter of patients and confirmed in half of these. When assessed, only one in three patients had elevated left atrial (LA) pressure [20]. It is likely that patients with presumably normal LA pressure could have been misdiagnosed with acute HFpEF or had echocardiography performed after normalization of LA pressure. In this series, patients were probably more often hospitalized for non-HF reasons. The Authors of this analysis concord on the fact that symptoms suggestive of acute HFpEF may in some patients represent non-HF comorbidities [20].

## 3. Age-Related Myocardial Remodelling

Aging of the myocardium is itself a specific pathophysiological process [21], representing a growing problem across developed countries. In fact, age is strictly related to the development of heart failure. Heart failure affects about 1% of subjects in their 50s and progressively rises with age to afflict 10% of persons in their 80s [22]. The increased incidence of HF with age is particularly evident in HFpEF [23]. There are of course several pathological reasons at the base of this association, since all conditions favouring the development of frank heart failure increase with age. However, apart from classic heart failure determinants, cardiac aging itself is a non-diagnosed condition. In the following paragraphs cellular, metabolic and molecular factors contributing to myocardial aging and potential failure will be discussed.

**Fibrotic cells are the wrinkles of the heart.** Organ fibrosis is a consequence of aging. Longitudinal clinical trials such as the Framingham Heart Study [23] and the Baltimore Longitudinal Study of Aging [24] have evidenced that aging is associated with LV hypertrophy. LV hypertrophy may be considered an adaptation to maintain normal systolic function with aging. This hypertrophy often affects the LV in an asymmetrical way, mostly affecting the subaortic segment of the ventricular septum and variously termed subaortic ventricular septal bulge (VSB), sigmoid-shaped septum and localized discrete upper septal hypertrophy [25]. A recent study has shown a direct relation between VSB and age, whereas no significant independent association of VSB with hypertension and other cardiovascular risk factors was found [26]. The presence of VSB was associated with enhanced global LV systolic function and some dilation of the aortic root. Despite no significant impact on exercise capacity was noticed after accounting for potential confounders, this structural cardiac adaptation could contribute to the development of fibrosis, arrhythmias, progressive myocardial deterioration, and end-stage heart failure [27].

However, before eventually developing overt chronic systolic heart failure, these elderly patients may well experience episodes of acute decompensation. Infarct, atrial fibrillation, uncontrolled chronic arterial hypertension, hypertensive crisis, acute infective diseases, myocardial ischemia, to mention the most frequent, can all determine the development of acute heart failure. Since all these conditions are very frequent in the elderly, and fibrosis is progressively demonstrated with age increase, this means that almost all the elderly have various degrees of HFpEF. Do we have to treat these patients specifically, or do we have to adopt specific therapies aimed at controlling the potential triggering factors? As cardiologists, and doctors in general, we should try to individualize the correct and appropriate therapy, especially in the coming era of precision medicine.

**Age-related reduction of cardiac cellular metabolic reserve.** In normal myocardium, the concentrations of the high-energy compounds adenosine triphosphate (ATP) and phosphocreatine (PCr) are firmly controlled because ATP production by mitochondrial oxidative phosphorylation is closely coupled to ATP utilization by cytosolic ATP. The latter is the direct energy source for energy-consuming reactions in the cell, while PCr acts as an energy storage compound and, in addition, as an energy transport molecule in the ‘creatine kinase-PCr energy shuttle’. PCr/ATP ratio is reduced in hypertrophied and in failing human myocardium [28]. In a recent study a possible relation between age and PCr/ATP ratio and cardiac power in healthy women by cardiac MRS with 31P spectroscopy and maximal cardiopulmonary exercise testing has been investigated [29]. PCr/ATP ratio, peak cardiac power, diastolic function and peak exercise oxygen consumption were significantly lower in the elderly compared with the young age group. PCr/ATP ratio showed a significant positive relationship with diastolic function, peak cardiac power output and peak oxygen consumption; resting and exercise systolic blood pressures were higher in the older age group. Since blood pressure is an important determinant of cardiac energy consumption, it could have well determined the observed reduction in PCr/ATP ratio in this group. These results indicate that high-energy phosphate metabolism and peak power of the heart decline with age. Additionally, based on the positive relationship between PCr/ATP ratio, early-to-late diastolic filling ratio and peak cardiac power output, they confirm that cardiac high-energy phosphate metabolism may be an important determinant of cardiac function and performance. This observation indicating that high-energy phosphate metabolism and performance of the heart decline with age and yield a positive relationship with peak cardiac power output suggests that reduced cardiac high-energy phosphate metabolism could play a significant role in the context of borderline cardiac conditions. PCr/ATP ratio in healthy young male adults is also inversely associated with heart rate, which is the other important determinant of cardiac energy consumption [30]. In fact, in the context of physiological/pathological conditions transiently determining increased myocardial demand (strenuous exercise, strong emotions, uncontrolled hypertension, fever, infections, etc), reduced cellular energy reserve in an apparently healthy heart (preserved ejection fraction) could well determine acute heart failure. On the other hand, could these people be labelled as having chronic heart failure (HFpEF)? Nevertheless, in this context, cardiac MRS with 31P spectroscopy for PCr/ATP ratio determination could represent a very useful tool to better characterize otherwise healthy elderly patients in terms of prognostic stratification [31]. However, aging is by itself an unsuccessful process. The principal physiologic characteristic of aging is a decrease of tissue reparative and regenerative capacities. The number of cardiomyocytes [32] and their renewal [33] decline progressively with aging. Cardiac troponin T levels increase with aging and can predict cardiovascular events and death in the general population [34]. This finding could be related to age-related reduction of expression or activity of proteins that are involved in cardio protection, a condition that eventually leads to an increased susceptibility of cardiac myocytes to injury [35].

**Are timely wrinkles pathological?** According to the World Health Organization, aging is a course of biological reality which starts at conception and ends with death. Aging determines physiological changes in all organ systems; age related cardiovascular changes occur in parallel with age related changes elsewhere in the body. The cardiac output decreases, blood pressure increases on a multifactorial basis and blood vessels become sclerotic. The lungs exhibit impaired gas exchange. The kidneys decrease their filtering function. Atrophic gastritis and altered hepatic drug metabolism are common in the elderly. Due to a progressive decline in bone mass after the fourth decade, osteoporosis is frequently observed. With age, skin atrophies due to changes in collagen and elastin content, losing its tone and elasticity. Lean body mass declines with age and this is primarily due to loss and atrophy of muscle cells. Degenerative changes occur in most joints and this, combined with the loss of muscle mass, reduces elderly locomotion. Metabolism is altered, changes in response to commonly used drugs make different drug dosages necessary. Overall, trying to simplify the concept, the principal age-related cardiovascular changes results from alterations of the connective tissue matrix, determining reduction in elasticity and distensibility of myocardial, vascular and valve structures. The conduction system is also involved by progressive fibrosis and loss of specialized cells. These processes appear particularly at work in the over 75 years group [36] and even though the individual aging process is probably genetically determined [37] rational preventive programs of diet and exercise have been designed to delay or reverse some of these changes [38]. Present and future science developments to delay the tissue aging process include approaches aimed at reducing oxidative stress [39], DNA damage [40], telomere shortening [41], advanced glycation end (AGE) products [42], chronic low-grade inflammation [43], and at improving noncoding RNAs regulation [44]. However, the philosophical approach is that at present we must deal with a condition we should continue to consider as physiological: tissue aging. Apart from the known risk factors which accelerate the process, cardiovascular tissue aging is certainly one of the main causes of HFpEF. Reduction of coronary blood flow and of diastolic, valvular and conduction system functions, all contribute to potential acute decompensation in concomitance of trigger events. Is there any possibility to find a single treatment for such a composite condition? It is very unlikely.

## 4. Age of Patients in Trials of HFpEF is Greater than Patients with HFrEF

Recently, a systematic search of HF trials enrolling more than 400 participants published between January 2001 and December 2016 using PubMed/Medline and ClinicalTrials.gov. has evaluated a total of 118 trials enrolling a cumulative 215,508 patients [45]. Trial findings were compared with large epidemiologic studies indexed to hospitalization status and ejection fraction. Overall, 94 trials (80%) enrolled patients with HFrEF exclusively. Age of trial participants was 65 + 11 years (from 64 years in 2001 to 2004 to 65 years in 2013 to 2016). HFpEF trials enrolled older participants mean age 71 + 7 years. Corresponding mean ages in US epidemiologic studies were 69 years for HFrEF and 73 years for patients with HFpEF.

HFpEF is mainly a disease of ageing and is associated with widespread vascular, arterial, venous and cardiac stiffening and other co-morbidities. HFpEF is particularly common among the elderly population, because even when healthy and normal, older persons have substantial limitations in cardiovascular reserve, including cardiac output, heart rate, stroke volume, systolic and diastolic function, compared to younger subjects [46, 47, 48].

## 5. Pathogenetic Factors in HFpEF

**Fig. 1.**
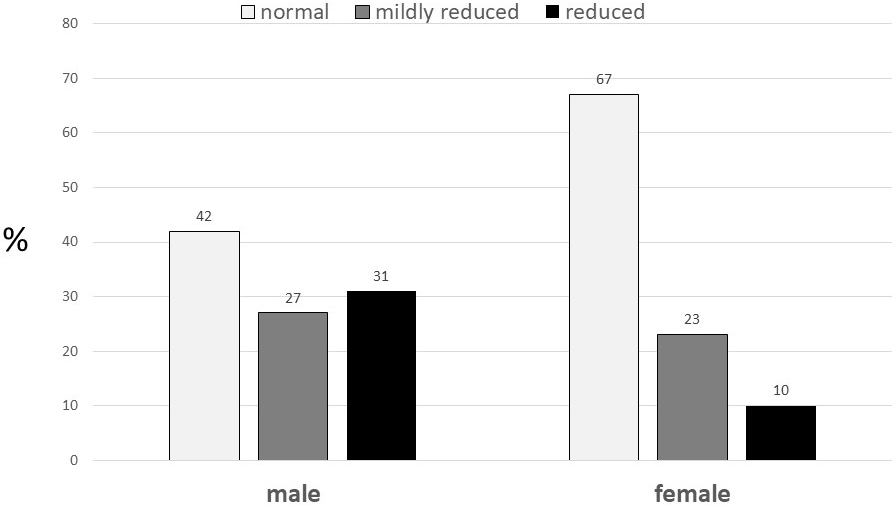
**Left ventricular ejection fraction by gender among participants with congestive heart failure in the Cardiovascular Health Study**. (From reference [54]).

**Female gender.** Epidemiological and registry studies show that women have an incidence of HFpEF like that of men [49, 50, 51, 52, 53]. In the PURSUIT-HFpEF prospective multicentre East-Asian HFpEF registry [53], women accounted for 55.2% of the overall cohort. The Cardiovascular Health Study Research Group observed that acute cardiac decompensation is common among community-dwelling elderly, it increases with age and is usually associated with normal systolic LV function, particularly among women (Fig. 1, Ref. [54]). Specific patterns of LV remodelling in women may depend on sex-specific cardiomyocyte loss, increase in extracellular matrix, and myocellular hypertrophy. Peculiar vascular stiffening, driven by changes in endothelial dysfunction, elastin–collagen content, microvascular function, and neurohormonal signalling may also be additional pathogenetic factors related to gender. Oestrogen is implicated as an important mediator of the above-mentioned changes and, due to its direct vasodilator activity, it promotes nitric oxide excretion and impacts myocellular ${\mathrm{Ca}}^{2+}$ handling, mitochondrial energy production and oxidative stress. These sex-specific cardiac and vascular changes may be determinant for heart failure development, particularly of the preserved ejection fraction variety. Again, future preventive strategies and therapeutic approaches should take these factors into account [55].

**Diabetes and obesity. **About 45% of patients with HFpEF have diabetes mellitus (DM), and the prevalence of comorbid DM is increasing most significantly in those with new-onset HFpEF [56]. A recent study has summarized data from several clinical trials that examined the effect of DM in HFpEF and provided previously unpublished data from a large cohort of HFpEF patients with and without DM [57]. All together data suggest that DM is associated with increased morbidity and long-term mortality in HFpEF. The Authors also point out several common pathological mechanisms in HFpEF and DM, including sodium retention, metabolic derangements, impaired skeletal muscle function, some of which could represent potential therapeutic targets, as discussed below.

Obesity is a major risk factor for HF [58]. Previous studies have clearly shown that obesity and weight gain promote abnormalities in myocardial structure and function implicated in the development of HFpEF [59, 60]. Patients with obesity related HFpEF show distinct pathophysiologic features including greater biventricular remodelling, volume overload, RV dysfunction, greater ventricular interaction and pericardial restraint, worse exercise capacity, more profound haemodynamic derangements, and impaired pulmonary vasodilation [61]. These features have suggested that obesity may be considered as a specific HFpEF phenotype and should be considered for potential therapeutic approaches [61]. However, the most obese patients continue to be excluded from HFpEF clinical trials, and thus ongoing research should try to determine the role of pharmacologic and interventional approaches in this growing population [62].

**Ischemic heart disease. **Ischemic heart disease (IHD) is a major pathogenic factor in HFrEF. Similarly, the prevalence of IHD in HFpEF has been estimated to range between 38 and 59%, the high variability due to patient characteristics or the actual definition of IHD and the type of HFpEF [63, 64, 65]. Huang and Colleagues have recently evaluated the clinical, structural, functional, and outcome characteristics in a group of 376 patients who were previously hospitalized for HFpEF [66]. Of the HFpEF patients who underwent coronary angiography they found that approximately two-thirds had coronary artery disease (CAD) (defined as >50% stenosis) and these patients had greater deterioration in ventricular function and increased mortality during follow-up compared with those without significant coronary lesions. In fact, patients with HFpEF and CAD appear to be at high risk of cardiovascular death [63]. Further confirmation of this finding comes from a recent post hoc analysis of TOPCAT (Treatment of Preserved Cardiac Function Heart Failure with an Aldosterone Antagonist Trial) study [65]. It shows that the prevalence of ischemic heart disease in patients with HFpEF was 59%, and that the participants with ischemic heart disease had a 20% higher risk of major adverse renal and/or cardiac events compared to those without. Factors including body mass index, smoking, diabetes mellitus and dyslipidaemia were also found to contribute to the risk of adverse events.

Apart from epicardial coronary stenoses, coronary microvascular dysfunction may promote cardiac injury by inducing myocardial supply-demand mismatch, especially during exercise, leading to systolic and diastolic reserve limitations, higher filling pressures during exercise, and more impaired exercise capacity [67]. Coronary microvascular dysfunction has been proposed to be a potential mechanism underlying the pathogenesis of HFpEF [68, 69, 70, 71, 72]. Coronary microvascular dysfunction is seen in about three quarters of patients with HFpEF in the absence of revascularized macrovascular CAD and has been shown to be associated with systemic endothelial dysfunction as well as markers of HF severity (NT-proBNP and RV dysfunction) [73].

The observed high prevalence of ischemia within the HFpEF population, as well as its association with increased risk of adverse events, clearly suggests the need to create specific interventions for this sub-population.

**Hypertension.** Hypertension is the putative father of HF [74]. This is why hypertension is managed so intensively in the clinical setting [75]. Despite aggressive treatment, almost all hypertensive patients develop some degree of myocardial dysfunction, once called diastolic dysfunction, now called HFpEF. The dysfunction degree depending on hypertension duration, patients age, ethnicity, dietary sodium and comorbidities, including obesity, diabetes mellitus, and chronic kidney disease [76, 77]. LV filling dynamics may be at work well before the development of LVH [78]. When these patients develop symptoms of heart failure, but with preserved systolic cardiac function, they are then classified as HFpEF. Potential triggers can cause acute decompensation of HFpEF and include uncontrolled hypertension, salt and fluid overload and, again, onset of atrial fibrillation, myocardial ischemia, progressive kidney disease, anaemia, chronic obstructive pulmonary disease and infections [79].

By using 140/90 mmHg or greater as diagnostic criteria, an estimated 1.13 billion people worldwide have hypertension [80]. By applying the American College of Cardiology/American Heart Association definition of hypertension as 130/80 or greater [81] an additional 30% of hypertensive subjects should be added. Epidemiologic data from the Framingham Study show that hypertension has the greatest impact in the population burden of heart failure among the modifiable risk factors that promote it, accounting for 39% of CHF events in men and 59% in women [82]. In the elderly population, as many as 68% of heart failure cases are attributed to hypertension [83]. In short, we are talking about around one billion people around the world, potentially carrying HFpEF according to present nosology. In fact, these patients must be effectively treated, with very beneficial effects especially in terms of new onset HF reduction [84]. Nevertheless, in such a wide population where multiple co-pathologies likely co-exist, the concerted therapy should be based on a pathophysiological approach [85]. This is sometimes difficult to achieve, since some of the ordinary drugs used in daily practice yield additional pharmacological actions, those primarily affecting global and cardiac metabolism deserving special attention [86]. In this context the idea of finding a unique treatment that would eventually reduce events in such a complex clinical situation appears rather as mere wishful thinking.

**Atrial fibrillation (AF).** Atrial fibrillation often coexists with HF; they are mechanistically linked to each other and can adversely impact cardiovascular outcomes and mortality. Data from the observational, prospective, HF long-term registry of the ESC show that AF is significantly associated with worse cardiovascular outcomes in patients with HFpEF and HFmrEF, but not in those with HFrEF [87]. The differential association of AF with adverse cardiovascular outcome between the EF subtypes might be that with higher EF, AF may contribute to progression of HF and worsen outcomes, whereas with lower EF, the HF disease itself determines the outcome [88, 89]. The notably greater role of AF in HFpEF may also be related to potential undertreatment and eventual lesser response to HF therapy.

It has also been hypothesized that the strict epidemiological and clinical parallelism of AF and HFpEF could be related to a potential common mechanistic substrate characterized by a systemic inflammatory or metabolic disorder, causing coronary microvascular dysfunction and fibrosis of the atrial and ventricular myocardium [90]. Alternatively, since AF and HFpEF share common risk factors, unmeasured confounders or not-well-documented risk factors may coexist and may confer excess mortality in patients with AF and HFpEF [89].

By the way, apart from lone AF in the young, according to the present nosology all patients with AF could be defined as also carrying HFpEF. Those patients with permanent AF are at risk of developing acute HF: they usually carry structural heart abnormalities (at least atrial enlargement), some promoting conditions (i.e., hypertension, diabetes, ischemic heart disease) and, in presence of triggering factors (i.e., uncontrolled heart rate, acute infections), they can acutely decompensate. So, are these patients in the hodgepodge of HFpEF already before acute decompensation, or do we have to wait for the first acute event? The lifetime risk of AF was estimated about 1 in 4 in white men and women older than 40 years in 2004 [90]; a decade later, lifetime risk estimates reached about 1 in 3 in white and 1 in 5 for black individuals [91, 92].

Again, it appears very difficult and over-ambitious to look for any specific treatment that could be effective to reduce events in all the various forms of AF. Atrial fibrillation is a specific condition, it really is a kind of “failure” of the heart, in the sense that it does deprive the circulation and the cardiac power of the atrial contribution to ventricular filling which, depending on comorbidities and clinical contexts, shall need specific treatments [93].

**Chronic kidney disease (CKD).** Chronic kidney disease is commonly observed among patients with HFpEF and is associated with the worst clinical outcomes [94, 95]. In most cases this association probably depends on the common pathophysiological milieu at the base of the two conditions: a greater hypertensive and/or atherosclerotic burden, a longer duration of the predisposing conditions [96]. In presence of CKD, whatever the reason, making renal function worse, can induce acute heart failure due to volume overload [97]. Pre-existent systolic and diastolic failure may be an aggravating factor, but they are not always necessary to produce acute cardiac decompensation. Nevertheless, even though CKD has been shown to be more common in HFpEF than in HFmrEF and HFrEF, it may have more of a bystander role in HFpEF, being less associated with mortality and with lower prognostic discrimination [98].

**HFpEF and restrictive cardiomyopathies. **Restrictive cardiomyopathies (RCM) are characterized by diastolic dysfunction due to infiltration of the myocardium or ventricular hypertrophy, resulting in increased myocardial stiffness and leading to impaired ventricular filling. Biventricular chamber size and systolic function are usually normal or near normal until later stages of the disease. Most restrictive diseases are represented by myocardial infiltration by other substances such as amyloid, iron or glycogen or endomyocardial fibrosis. The principal infiltrative cardiomyopathies include cardiac amyloidosis, sarcoidosis, hemochromatosis and Fabry disease. RCM may cause left or right heart failure by affecting either or both ventricles, Arrhythmias and conduction disturbances are frequently encountered [99]. As the presentation is nonspecific, rapid recognition of RCM is challenging. Patients frequently undergo extensive cardiac evaluation, including coronary angiography, without a diagnosis. Then, many patients may be misdiagnosed and labelled as having HFpEF. Indeed, these patients have normal systolic function, may undergo acute cardiac decompensation, especially in the context of concomitant pathologic conditions and should deserve a proper diagnosis. Interestingly, a recent report suggests that 15–20% of patients with HFpEF may have amyloidosis and this high percentage of patients is often non diagnosed [100]. In fact these patients are affected by a specific disease [101], they carry a poor outcome and unsurprisingly may not respond to conventional treatments of HF, unless they are treated with the upcoming and specific treatments, including therapies designed to directly target myocardial deposits or replace enzymatic defects, whenever feasible and effective. When a diagnosis is accurate and made in a timely manner, a patient has the best opportunity for a positive health outcome because clinical decision making will be tailored to a correct understanding of the patient’s specific health problem [102].

**Heart failure is not only the left ventricle: role of right ventricular function. **It is implicit that when we talk about HFpEF, the term preserved is limited to the LV. Nevertheless, RV dysfunction (RVD) and pulmonary hypertension (PH) are increasingly recognized in patients labelled as HFpEF. Both conditions are associated with poor outcome in patients with HFpEF [103, 104]. The reported prevalence of clinical RVD in HFpEF ranges between 4% and 50%, partly due to lack of consensus on its definition but also as the result of heterogeneity of the studied populations [103, 104, 105, 106]. The aetiology of HFpEF in RVD is likely multifactorial. Treatment aimed at improving RV performance and PH in HFpEF has proven unsuccessful thus far, compelling the need for evaluation and discovery of novel therapies for this commonly occurring concurrent condition [107, 108]. However, the concept of HFpEF and RVD is a difficult one to accept, and this the most evident mistake at the base of the whole lot: we are in the presence of a distinct pathological entity (i.e., RVD) and yet we refer to the left ventricle (HFpEF). Why? Right ventricular dysfunction in an autonomous entity, which has its own pathophysiology, diagnosis, treatment and prognosis.

**Potential additional factors related to occurrence and outcome in heart failure with a preserved ejection fraction. **As described above, several comorbidities may dictate prognosis and outcome in HFpEF [109]. Apart from those principal fields outlined above, there is a huge bulk of references regarding additional comorbidities that can be responsible for the onset and progression of HFpEF. The scope of this paper is just to re-discuss the appropriateness of the present nomenclature, in order to eventually re-direct the efforts aimed at finding specific treatments for such a complex condition. However, for the sake of completion, all potential additional factors which have been associated with the pathogenesis of HFpEF will be mentioned and partially referenced in the following lines, in random order: anaemia [110], thyroid dysfunction (both hypo- and hyperthyroid disease) [111], obstructive sleep apnoea [112], chronic pulmonary disease [108], sarcopenia [113], stroke [114], peripheral arterial disease [115]. Recently, it has been confirmed that a greater burden of non-cardiac organ dysfunction, sedentariness and functional impairment distinguish patients with HFpEF and prior HF hospitalization from those never hospitalized [116].

Finally, a very recent study has confirmed that diffuse myocardial fibrosis, and hence, possibly, HFpEF [117], are frequently observed in asymptomatic patients with valvular heart disease and preserved LV systolic function. According to these results, myocardial fibrosis is present at an early stage of the disease, well before developing detectable LV dysfunction and symptoms. However, since the relationship between the progressive magnitude of myocardial fibrosis and potential prognostic implications are not yet defined, further studies on this topic are warranted.

**Fig. 2.**
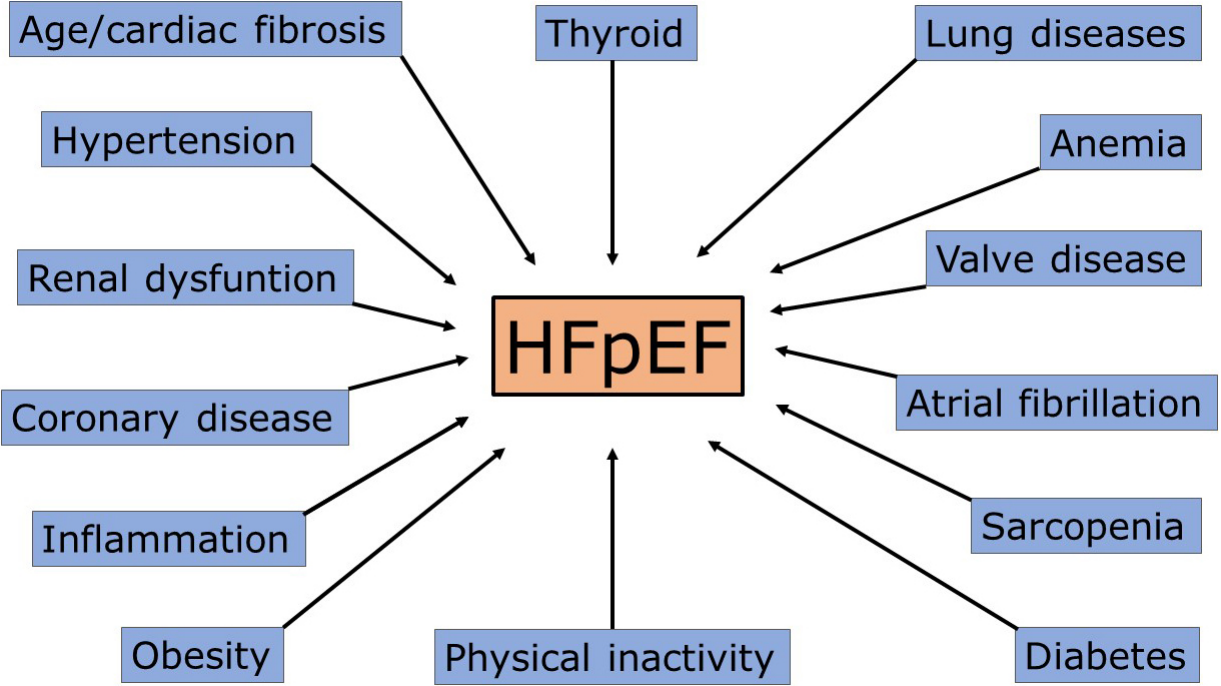
**Principal mechanisms/causes of heart failure with preserved ejection fraction (HFpEF)**. Each cause could determine acute cardiac decompensation, especially in overlap conditions.

**The actual potential size of HFpEF. **Based on the present classification, the number of patients potentially entering the HFpEF pot could be extremely high. By considering some of the main pathology clusters discussed above (Fig. 2), the count could be a considerable fraction of potentially affected patients worldwide: a fraction of 1 billion people with hypertension [118], 300 million with diabetes [119], 2 billion with overweight/obesity [120], 700 million with coronary disease [121], 700 million with chronic pulmonary disease [122], 700 million aged >65 year [123], 37 million with atrial fibrillation [124], all patients with potential HFpEF. These figures confirm the need for reappraising such classification.

## 6. HFpEF Clinical Phenotypes

As delineated above, rather than a distinct pathophysiologic entity, the syndrome of HFpEF encompasses a heterogeneous group of patients with a wide range of factors that contribute to HF pathophysiology. Several studies have attempted to subclassify HFpEF into more homogenous subgroups. Kao *et al*. [125] presented an exploratory study of patients enrolled in the Irbesartan in Heart Failure with Preserved Ejection Fraction Study (I-PRESERVE) and identified six subgroups of HFpEF patients with significant differences in event-free survival. The worst event-free survival were characterized by a subgroup with high prevalence of obesity, hyperlipidaemia, diabetes mellitus, anaemia, and renal insufficiency and by another subgroup with female predominance, advanced age, lower body mass index, and high rates of atrial fibrillation, valvular disease, renal insufficiency, and anaemia [125]. Hedman *et al*. [126] used machine learning to identify six composite pheno-groups with significant differences in the prevalence of comorbidities (atrial fibrillation, chronic kidney disease, and anaemia), age, gender, clinical and laboratory variables, cardiac structural and functional alterations. These pheno-groups were characterized by differential levels of inflammatory and cardiovascular proteins, and outcomes [126]. However, in this study of European patients with HFpEF a pheno-group of obese patients was not observed, as previously found in an US setting [127].

More recently, from the Swedish Heart Failure Registry a cluster model from 6909 HFpEF patients was derived [128] and allowed for a novel classification technique to identify clinical phenotypes. Overall, heterogeneity of HFpEF was hefty and this technique was able to identify five distinct clinical clusters of patients: a young-low comorbidity burden cluster, an atrial fibrillation-hypertensive cluster, an older-atrial fibrillation cluster, an obese-diabetic cluster, and a cardio-renal cluster. Patients in the young-low comorbidity burden cluster had the lowest, while those in the older-atrial fibrillation and cardio-renal cluster had the highest event rates.

Overall, these studies confirm that in HFpEF, cardiac and extracardiac comorbidities such as aging itself, arterial hypertension, atrial fibrillation, diabetes and obesity, ischaemic heart disease, renal insufficiency, lung conditions and presence of right-sided HF as well as female gender play important roles in defining presence of HFpEF, its cause, pathophysiological mechanisms, outcomes, and eventual treatment approaches. The opinion is that we should not talk about specific phenotypes in this syndrome. Different pathologic conditions alone or in combination are the cause of acute decompensation episodes. The search for different phenotypes characterizing HFpEF appears futile and confirms the importance of better defining the pathological conditions characterizing the single patient, in various combinations, not necessarily being interconnected.

## 7. Therapeutic Implications

**Fig. 3.**
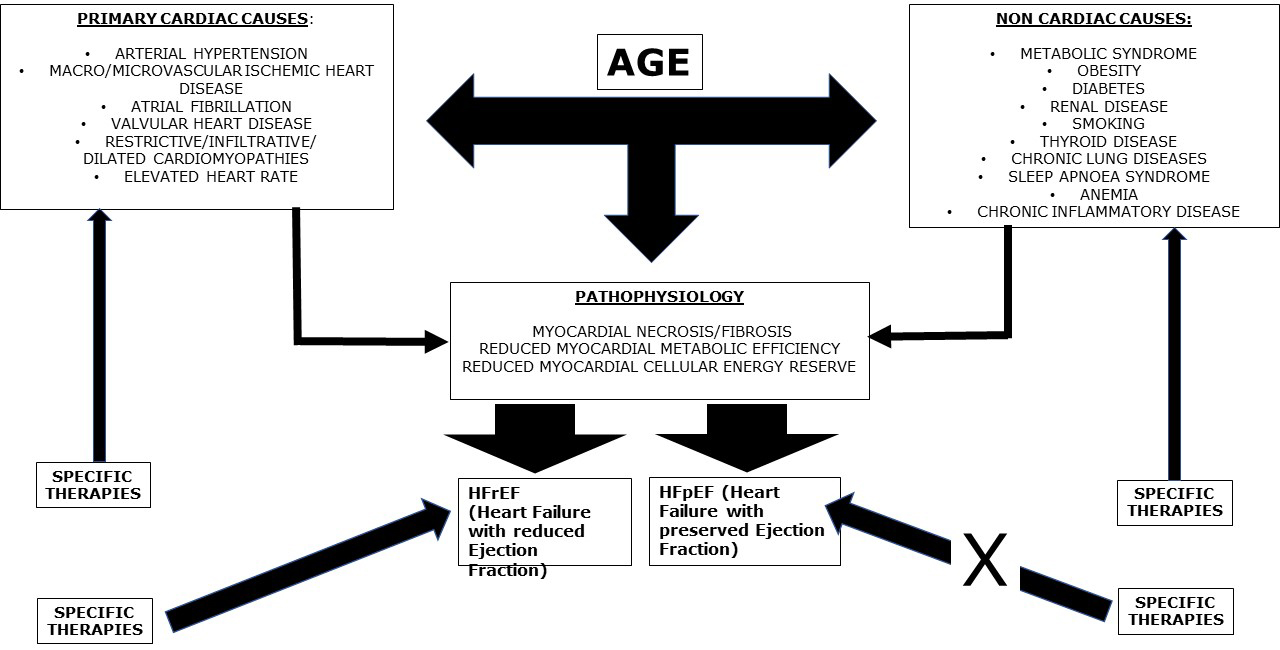
**The development of heart failure (HF) is due to primary cardiac causes which can, per se, directly determine HF with reduced ejection fraction (HFrEF), or eventually preliminarily pass through the condition of minimal myocardial damage, not determining significant left ventricular dysfunction and, therefore, entering in the classification of HF with preserved ejection fraction (HFpEF)**. Non cardiac causes alone generally determine HFpEF, which can eventually progress towards HFrEF, especially if associated to a cardiac cause: for example, a diabetic patient with an acute coronary syndrome. Age is a main regulator, for obvious reasons, playing a specific and mostly autonomous role in the development of HF. Whatever the cause of HF, age is a common aggravating factor. Age carries the condition of HF. Overall, specific therapies are aimed at preventing major cardiac causes of HF. They can also efficiently counteract all the additional promoting factors for heart failure, therefore reducing their impact in the pathogenesis of cardiac damage. Specific therapies are also effective in reducing the burden of HFrEF, while up to now they have been ineffective in reducing that of HFpEF, probably because the neurohormonal activation in the latter is not yet considerable.

Despite the scope of a therapy would ideally be the discard of the causative agent, this goal is often unachievable for different reasons related to specific diseases (Fig. 3). This is particularly relevant in HFrEF. The damage is often irreversible, the main ambition is to prevent acute decompensation episodes and mortality. A previous cardiomyocyte injury determining LVEF below 40% triggers neurohormonal mechanisms to maintain the cardiac index and organs perfusion. The “neurohormonal hypothesis” (abnormal activation of the sympathetic nervous system [SNS], renin-angiotensin-aldosterone system [RAAS]) has been key to understand the pathophysiology of HFrEF. Building on this knowledge, several trials have established the efficacy of neurohormonal antagonist drugs (i.e., antagonization of SNS by beta-blockers) in patients with LVEF <40%, yielding a tremendously favourable outcome, even in presence of coexisting diseases in which the same therapies could be contraindicated. Pharmacological inhibition of neurohormonal activation response to heart failure is a way to reduce a physiological adaptative response which was probably effective when mankind was principally dependent on daily personal performance. No hunting, no dinner. In war times there is not much fuss about long term prognosis: the fight is for day-to-day survival. Indeed, in the present era we are more organized to accept the reversal of such short-term adaptive response, avoiding the progression toward maladaptation.

Betablockers. In HFrEF this class of drugs remains effective, despite many patients often carry other conditions where betablockers are traditionally considered contraindicated. In fact, in HFrEF sustained activation of endogenous neurohormonal systems in response to impaired cardiac pumping greatly benefits from long term therapy with beta-blockers, associated with evidence of decreased plasma markers of activation of the sympathetic nervous system, the renin-angiotensin system, and endothelin-1. This pathophysiological pharmacologic effect may be enough to overcome potential unwanted effects of these drugs (for example negative inotropism, negative effects on glucose and lipid metabolism). Several clinical trials have assessed whether the therapeutic paradigms obtained by HFrEF studies could be translated to patients with HFpEF. Indeed, trials have consistently shown that beta blockers do not significantly improve patient prognosis [129, 130]. This systematic failure of clinical trials on drugs for neurohormonal antagonism in HFpEF is likely due to the much lower neurohormonal activation observed in these patients, as compared to patients with HFrEF [131]. Sympathetic activation is significantly associated with all-cause and cardiovascular mortality across the entire LVEF spectrum [131]. Interestingly, the association between sympathetic activity and cardiovascular mortality according to LVEF categories has shown the strongest association in the group of patients with HFmrEF and the weakest in HFpEF [132]. This fact could help to explain why the response to sympathetic antagonism in patients with HFmrEF is similar to HFrEF, rather than to HFpEF. When systolic function decreases, sympathetic function correspondingly increases and then, only then, relative antagonism to this pathophysiologic adjustment becomes useful.

Renin-angiotensin-aldosterone system (RAAS) inhibitors and angiotensin receptor neprilysin inhibitors (ARNI). Table 1 (Ref. [133, 134, 135, 136, 137, 138, 139, 140, 141, 142, 143]) summarizes the principal studies. Individual studies have failed to demonstrate significant benefits of these classes of drugs in patients affected by HFpEF [133, 134, 135, 136, 137, 138, 139, 140]. Systematic reviews and pooled analysis have also failed to find any significant positive effects on mortality [144, 145, 146, 147]. The heterogeneity in the enrolled populations may have influenced the results of these studies. Indeed, HFpEF patients’ mortality is usually influenced by age and associated comorbidities, such as renal dysfunction, respiratory diseases and diabetes, rather than progression of HF, arrhythmias and other cardiac causes [148, 149]. Comorbidities should be regarded as key therapeutic targets and objects of dedicated quality improvement initiatives, especially in patients with no or mild systolic dysfunction [147]. In this context, Cohen and Colleagues [150] among TOPCAT [139] participants have tried to identify HFpEF phenogroups based on standard clinical features and assessed differences in multiple biomarkers, cardiac and arterial structure/function, prognosis and response to spironolactone. The results of this analysis show three distinct clinically identifiable phenogroups: phenogroup 1 (“Younger with mild symptoms”), phenogroup 2 (“Older with stiff arteries, small LV and atrial fibrillation”), and phenogroup 3 (“Obese, diabetic with advanced symptoms”). The two latter phenotypes, which constitute genuine high-risk HFpEF, exhibit distinct abnormalities in biomarkers, cardiac/arterial structure and function, and differential response to spironolactone therapy. In contrast, Phenogroup 1 represents a low-risk group which may not represent genuine HFpEF and may be confounded by lung disease, which in turn explains geographic differences in TOPCAT [150].

**Table 1.** **Principal studies employing renin-angiotensin-aldosterone system (RAAS) inhibitors and sodium–glucose co-transporter 2 inhibitors (SGLT2i) in patients with HFpEF**.

	Intervention	Eligible	Primary composite	Treatment effect	Overall efficacy
		LVEF	endpoint	(primary endpoint)	
CHARM-preserved	ARB	>40	CV death or HFH	Unadjusted HR: 0.89;	Neutral,
n = 3023	(candesartan)		(time to first)	*p = *0.118	reduction in HFH
Ref [133]				Covariate adjusted HR: 0.86;	
				*p = *0.051	
PEP-CHF	ACE-i	>40	All-cause of death	HR: 0.919; *p* = 0.545	Neutral
n = 850	(perindopril)	(wall motion	or HFH (time to first)		
Ref [134]		index <1.4)			
I-Preserve	ARB (irbesartan)	>45	All-cause death	HR: 0.95; *p* = 0.35	Neutral
n = 4128			or CV hospitalization		
Ref [135]					
Aldosterone antagonists and outcomes in real-world older patients with heart failure and preserved ejection fraction	MRA	≥40%	All-cause mortality	HR: 0.97; *p = *0.628	Neutral
n = 974			or HF hospitalization		
Ref [136]					
Association between use of renin-angiotensin system antagonists and mortality in patients with heart failure and preserved ejection fraction	RAS antagonists	≥40	All-cause mortality (assessed in a cohort matched 1:1 based on age and propensity score and in the overall cohort with adjustment for propensity score as a continuous covariate)	HR: 0.91; *p* = 0.008;	Positive
n = 16,216				HR: 0.90; *p* = 0.001	
Ref [137]					
Aldo-DHF	MRA	≥50	Changes in diastolic	Adjusted mean differences:	Positive;
n = 422	(spironolactone)		Function (E/e’)	–1.5 *p * < 0.001	no significant
Ref [138]			and maximal exercise	+0.1 mL/min/kg	change for peak
			capacity (peak ${\mathrm{VO}}_{2}$)	*p* = 0.81	${\mathrm{VO}}_{2}$
TOPCAT	MRA	≥45	CV death,	HR: 0.89; *p* = 0.14	Neutral
n = 3445	(spironolactone)		RSD,		
Ref [139]			or HFH		
			(time to first)		
PARAGON-HF	ARNI (sacubitril/	>45	CV death and total HFH (first and recurrent)	Rate ratio: 0.87; *p = *0.06	Borderline,
n = 140	valsartan)				favorable results
Ref [140]					in EF <57%
EMPEROR-preserved	SGLT2i	>40	CV death or HFH	HR: 0.79; *p * < 0.001	Positive
n = 5988	(empagliflozin)				
Ref [141]					
DELIVER	SGLT2i	>40	CV death or	HR: 0.82; *p * < 0.001	Positive
n = 6263	(dapagliflozin)		worsening HF		
Ref [142]					
PRESERVED-HF	SGLT2i	>45	Improvement	Effect size: 5.8 points;	Positive
n = 324	(dapagliflozin)		of KCCQ-CS	*p = *0.001	
Ref [143]					

ACE-i, angiotensin-converting enzyme inhibitor; ARB, angiotensin II receptor blocker; ARNI, angiotensin receptor neprilysin inhibitor; CHARM-Preserved, Candesartan Cilexetil in Heart Failure Assessment of Reduction in Mortality and Morbidity; CV, cardiovascular; DELIVER, Dapagliflozin Evaluation to Improve the Lives of Patients With Preserved Ejection Fraction Heart Failure; EMPEROR-Preserved, EMPagliflozin outcomE tRial in Patients With chrOnic heaRt Failure With Preserved Ejection Fraction; HF, heart failure; HFH, heart failure hospitalization; HR, hazard ratio; I-Preserve, Irbesartan in Heart Failure With Preserved Systolic Function; KCCQ-CS, Kansas City Cardiomyopathy Questionnaire Clinical summary; LVEF, left ventricular ejection fraction; MRA, mineralocorticoid receptor antagonist; PARAGON-HF, Prospective Comparison of ARNI With ACEI to Determine Impact on Global Mortality and Morbidity in Heart Failure; PEP-CHF, perindopril in elderly people with chronic heart failure trial; RAS, Renin-Angiotensin System; RSD, resuscitated sudden death; SGLT2i, sodium–glucose co-transporter 2 inhibitors; TOPCAT, Aldosterone Antagonist Therapy for Adults With Heart Failure and Preserved Systolic Function; HFpEF, heart failure with preserved ejection fraction.

Nitrates. The Nitrate’s Effect on Activity Tolerance in Heart Failure with Preserved Ejection Fraction (NEAT-HFpEF) trial compared the effect of isosorbide mononitrate or placebo on daily activity in 110 patients with HFpEF [151]. Patients on isosorbide mononitrate did not show better quality of life or submaximal exercise capacity as compared to patients on placebo.

Phosphodiesterase-5 Inhibitors. The Phosphodiesterase-5 (PDE-5) Inhibition to Improve Clinical Status and Exercise Capacity in Heart Failure with Preserved Ejection Fraction (RELAX) trial tested the hypothesis that therapy with the PDE-5 inhibitor sildenafil would improve exercise capacity in HFpEF assessed by the change in peak oxygen consumption [152]. After 24 weeks of therapy, changes in peak oxygen consumption in patients who received placebo or sildenafil were not significantly different.

Soluble guanylate cyclase stimulators. In HFpEF, endothelial inflammation leading to reduced nitric oxide bioavailability is hypothesized to culminate in decreased production of cyclic guanosine monophosphate (cGMP) by soluble guanylate cyclase (sGC) [71]. This pathway is involved in the regulation of myocardial contractility and relaxation, and impairments of this pathway have been associated with ventricular stiffening and hypertrophy, vascular stiffening and inflammation [153].

Two recent trials evaluated the use of direct sGC stimulators, vericiguat in VITALITY-HFpEF [154] and praliciguat in CAPACITY-HFpEF [155], to increase cGMP in patients with HFpEF. In the VITALITY-HFpEF trial [154], 789 patients with chronic HFpEF and LVEF of at least 45% with NYHA class II-III symptoms, within 6 months of a recent decompensation and with elevated natriuretic peptides were randomized to receive vericiguat or placebo. 24-week treatment with vericiguat as compared with placebo did not improve the primary outcome of change in the Kansas City Cardiomyopathy Questionnaire (KCCQ) physical limitation score. In the CAPACITY-HFpEF trial [155], 181 patients with HF and an EF of at least 40%, impaired peak ${\mathrm{VO}}_{2}$, and at least 2 conditions associated with nitric oxide deficiency (diabetes, hypertension, obesity, or advanced age), were randomized to receive 12 weeks of treatment with 40 mg of praliciguat daily or placebo. There was no significant difference in the primary outcome of change in peak ${\mathrm{VO}}_{2}$ from baseline to week 12, in the 40-mg praliciguat group compared with the placebo group. The results of these two trials do not support the use of soluble guanylate cyclase stimulators in patients with HFpEF.

Sodium–glucose co-transporter 2 inhibitors (SGLT2i). Table 1 includes the principal studies employing SGLT2i in HFpEF. In the recent years SGLT2i, a new class of glucose-lowering agents, have demonstrated cardiovascular safety in patients with type 2 diabetes mellitus (T2DM). Furthermore, some of these agents have been proven to have beneficial effects in reducing both major adverse cardiovascular events, cardiovascular mortality as well as hospitalisation for HFrEF. Based on published literature, the European Society of Heart Failure has recently issued a position paper stating that canagliflozin, dapagliflozin, empagliflozin, or ertugliflozin are recommended for the prevention of HF hospitalization in patients with T2DM and established at high CV risk of cardiovascular disease. Dapagliflozin or empagliflozin are also recommended to reduce the combined risk of HF hospitalization and cardiovascular death in symptomatic patients with HFrEF already receiving guideline-directed medical therapy, regardless of the presence of T2DM [156]. The recent adjourned guidelines of the ESC have confirmed these recommendations [157]. A recent study in patients with HFpEF, the Emperor-Preserved study, has shown that treatment with empagliflozin led to a lower incidence of hospitalization for heart failure, but it did not appear to affect the number of deaths from cardiovascular or other causes [141]. Interestingly, the benefit on total heart failure hospitalizations was similar in patients with an ejection fraction of >40–<50% and 50–<60% but was attenuated at higher ejection fractions. A similar dissociation between treatment effects on hospitalizations for heart failure and cardiovascular mortality was also seen in a previous trial with sacubitril–valsartan, which was conducted in a comparable patient population that was followed for a similar period [140]. In fact, the population enrolled in the Emperor-Preserved study is the mirror of what has been discussed in the present article: patients with too many variables, encompassing almost the complete cardiac pathology. In the Emperor-Preserved study, of recruited patients 35% had atrial fibrillation, 49% diabetes mellitus, 50% chronic kidney disease, 40% coronary artery disease, 14% anaemia, 13% chronic obstructive coronary disease, 8% pacemaker. Despite 85% of patients were reported to be on betablocker therapy, mean heart rate was still 70 beats per minute. Unsurprisingly in this mix-up of patients empagliflozin did not reduce mortality. Finally, an additional study employing dapagliflozin has demonstrated a reduction of the combined risk of worsening heart failure or cardiovascular death among patients with HFpEF [142]. Again, no changes in total mortality could be observed. The reduction of hospitalization could be just related to the diuretic effect of this class of drugs, but other mechanisms, not yet clearly defined, may also be involved. In fact, in the PRESERVED-HF study 12 weeks of dapagliflozin treatment significantly improved patient-reported symptoms, physical limitations and exercise function [143].

Addressing specific comorbidities. Several studies have shown that exercise training improves symptoms, exercise capacity, and quality of life in older patients with established HFpEF [158]. Anaemia correction, thyroid dysfunction correction, restoration of normal sleep breathing [159] have also been shown to improve prognosis in HFpEF.

HFpEF is determined by a myriad of concomitant, often overlapping, conditions, that it is worth to mention again: coronary artery disease, arterial hypertension, pulmonary disease, diabetes mellitus, obesity, anaemia, obesity, renal disease, sleep-disordered breathing, atrial fibrillation. Thereby, adopted therapies should always consider these (co)-pathologic conditions which, in the context of HFpEF often represent the main causative problem and should, therefore, be specifically targeted. In order to advance a more targeted approach to HFpEF classification and treatment, the implementation of precision medicine will better enable more targeted and more efficacious treatments to ameliorate the challenging HFpEF syndrome [160, 161].

## 8. Precision Medicine and Personalized Therapy

Precision medicine is a medical model that recommends individualized healthcare delivery in terms of medical decisions and treatments tailored to the single patient, instead of a one-drug-fits-all model. Artificial intelligence and machine learning are emerging as new tools toward precision cardiovascular medicine [162]. However, the personalization of medical therapies depends upon the understanding of the complexities of a biological system. Instead of developing treatments for populations and making the same medical decisions based on a few similar physical characteristics among patients, medicine is shifting toward prevention, personalization, and precision. In this cultural transformation, artificial intelligence is the key technology that can bring this opportunity to everyday practice [163]. In this context, it is surprising that a cultured community such as the cardiological one could put so much effort in the attempt to find a single effective treatment for a complex condition such as the so called HFpEF. Before using artificial intelligence we need human intelligence.

## 9. Conclusions

Nosology is the branch of medical science dealing with the classification of diseases. An accurate disease classification system is increasingly necessary to track the delivery of medical care and make decisions that can impact millions of individuals. A formalized nomenclature is essential for clear communication and to ensure that the classification system properly reflects advances in our understanding of disease mechanisms. This is especially true in the coming era of precision medicine, where specific treatments are more and more directed towards increasingly specific diseases. In this context, the identification of such a wide clinical syndrome, such as HFpEF, appears rather out of time.

Patients labelled as having heart failure with preserved ejection fraction are frequently elderly physically deconditioned subjects, especially women, with hypertension, obesity, glucose intolerance/diabetes, atrial fibrillation, anaemia, coronary artery disease, chronic pulmonary disease, and chronic renal insufficiency, alone or in combination. In practice, these conditions represent a large number of cardiac diseases we deal with in our daily clinical practice. For this reason, as already previously stigmatized [164], the HFpEF disease does not exist as a single entity and, as such, no specific unifying therapy could be found. New classification attempts still do not consider different phenotypes within the syndrome of HF and appears rather as an artefactual attempt to categorize a condition which is indeed not categorizable. Considering the huge economic efforts employed up to date to run very expensive trials and research in this field, it is time to call action to redirect such resources towards more specific pathophysiological classifications and potential specific therapeutic targets.
